# Giant Merkel cell carcinoma of the eyelid: a case report and review of the literature

**DOI:** 10.1186/1477-7819-9-58

**Published:** 2011-05-24

**Authors:** Luxia Chen, Limin Zhu, Jianguo Wu, Tingting Lin, Baocun Sun, Yanjin He

**Affiliations:** 1 TianJin Medical University Eye Center, 300084 TianJin P.R. China; 2 Department of Pathology of TianJin Medical University, TianJin Cancer Hospital, 300060 TianJin P.R. China

**Keywords:** Merkel cell carcinoma, eyelid tumor, diagnosis, histopatholog

## Abstract

Merkel cell carcinoma (MCC) is a rare cutaneous tumor and cases located in the eyelid have been described, but still its rarity may lead to difficulty in diagnosis and delay in treatment. A 51-year-old female patient that presented with large lesions in the eyelid underwent surgery after the diagnosis of acute chalazion. Following respiratory distress secondary to pulmonary metastasis, the patient's condition deteriorated and was not fit for complete excision treatment. Histopathological investigation of the biopsies, taken from the tumor, revealed that it was undifferentiated small cell carcinoma. Our aim with this paper is to point out that more cases should be reported for more effective diagnosis, histopathological study, clinical investigation, treatment and prognosis of this specific neoplasm.

## Background

Merkel cell carcinoma (MCC), sometimes referred to as a neuroendocrine carcinoma of the skin, arises from the uncontrolled growth of Merkel cells in the skin. It was first described by Toker [1] and since then many cases have been reported. To the best of our knowledge, involvement of the eyelid and face by large MCC has never been reported in the literature [2]. We here report a further case of the unusual tumor in the eyelid with histological, pictorial and immunohistochemical studies, which supports the hypothesis that it is derived from Merkel cells. We consider the histopathological diagnosis of mass in the eyelid to be very important. And diagnosis and treatment approaches of this entity are complex and require a skilled and experienced multidisciplinary team.

## Case Presentation

**Figure 1:**
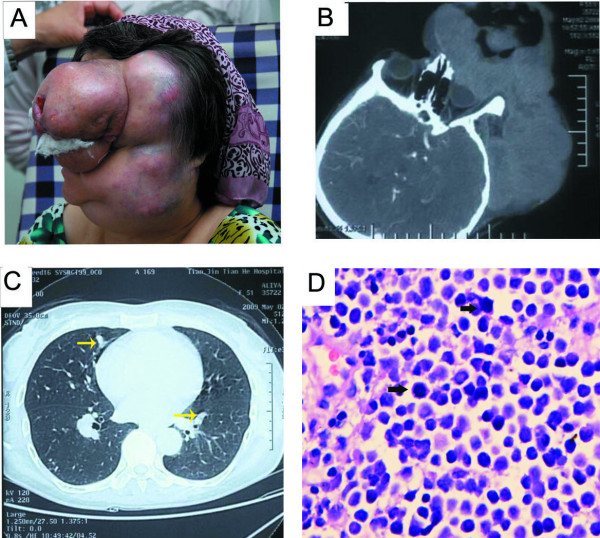
**Photograph showing patient who had a red lesion of the upper eyelid, the most common localization of ocular Merkel cell carcinoma, but the large lesion was uncommon**. (A) Bottom: (lateral view) The large violaceous mass that involves the entire left eyelid and facial surface multiple, ectensive large ulcer. The large tumor with multiple big dome-shaped nodules obscure boundary, plentiful blood vessels in the surface. (B) CT (computed tomography) scans show a large medium to high reflectivity mass. (C) CT showed that there were tumor metastases of mediastinal lymph node and multiple micrometastases (yellow arrow) of the lungs. (D) MCC with the mitotic index was high (black arrows) as stained by hemotoxylin & eosin.

A 51-year-old white woman was referred to ophthalmology centre at Tianjin Medical University with an enormous tumor mass on her left upper eyelid that was growing rapidly. General medical history revealed that the patient had been diagnosed with chalazion 3 years ago and was being treated with removal of the chalazion. Ophthalmic history was unremarkable and specifically there was no previous trauma. According to the patient and her family, the lesion first appeared on her left upper eyelid. On examination a firm lesion of the left eyelid measured 0.5 cm × 0.3 cm. Her physician initially diagnosed a chalazion and the patient was treated with incision of chalazion. One year later the cystic lesion had recurred and occupied half of the eyelid, measuring 1 cm × 0.6 cm, a fast-growing asymptomatic lesion in the same location with sinuous blood vessels covering its surface. But on her next visit three years later the tumor lesion was even larger, with necrotic and ulcerated areas on the surface, enlarged lymph nodes in the left cervical part. Examination revealed a large hard and poorly defined tumor, measuring 20 cm × 15 cm on its basal diameter and 10 cm in height with diffuse indurations of her left eyelid on which multiple, extensive large ulcer, big dome-shaped nodules could be seen (Figure 1A). The clinical presentation to the ophthalmologist and oncologist, a pate computed tomography (CT) scan suggested a superior eyelid mass lesion and enophthalmos (Figure 1B). Magnetic resonance imaging showed no invasion in orbit, but the results were compatible with a malignant eyelid. Further investigation revealed systemic metastasis. A chest CT scan showed multi-metastases in the apex of lung, metastasis mass of mediastinal lymph node and mediastinal lymphadenovarix (Figure 1C).

**Figure 2:**
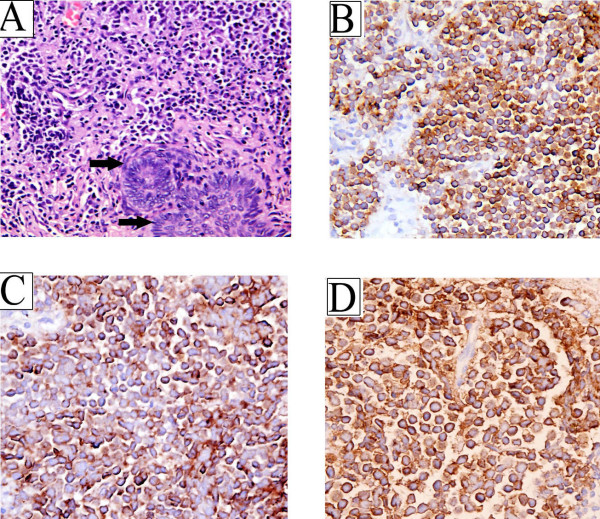
**Microscopic analysis of biopsy of Merkel cell carcinoma**. (A) Photomicrograph showing that Merkel cell carcinoma tumor cells are surrounded by intense inflammation with lymphocytes, plasma cells, and histiocytes. Proliferation of basophilic cells with round uniform nuclei, scanty cytoplasm, patchy chromatin and inconspicuous nucleoli (black arrows). (H&E, ×400). (B) Photomicrograph showing the same tumor stained for CK20. There is strong expression of CK20 in the cytoplasm and membrane of MCC. **C **Immunostaining with CAM5.2 showing characteristic para-nuclear accentuation. (D) NSE positive suffusion expresstion was localized on in the cytoplasm and membrane. (IHC, ×400).

In view of the suspected diagnosis of large malignant tumor, a biopsy was taken to confirm a provisional diagnosis. A biopsy was performed under local anaesthesia. Histopathological examination of the biopsy sample showed a tumoral infiltration of the dermis by rounded monomorphic cells of medium size with scant cytoplasm, round nuclei, and small nucleoli, clumps of a small cell tumor, forming solid masses or small trabecular structures. The tomor cells with the mitotic index was high (Figure 1D). The cells were arranged in large nests, masses, and strands (Figure 2A). The formation of glandular lumens was not observed. The tumor tissue immunohistochemical study proved positive for cytokeratin 20(CK20), neuronal specific enolase (NSE) and cytokeratin CAM5.2. The positive results are shown in Figure 2 (2B-D). There was no immunoreactivity to protein S-100, thyroid transcription factor 1(TTF-1) and leukocyte common antigen (LCA). Immunohistochemical staining showed characteristic. All these features above are consistent with the diagnosis of MCC. A diagnosis of MCC was made and the patient was referred to the Oncology Department. The patient's condition deteriorated rapidly with a midrange anaemia and she required palliative care for disseminated MCC by her oncologist.

## Discussion

Merkel cell carcinoma is a frequently lethal skin cancer that has a high propensity for nodal metastases and local recurrence, has poor prognosis. Several reports have described the association of MCC of the eyelids [3-5]. We report the case of MCC that the patient had been diagnosed with chalazion 3 years ago in the left upper eyelid and was being treated with surgical treatment. Although misdiagnosis of MCC pathologically as chalazions is a pitfall, this sometimes occurs. Lesions demonstrate a broad spectrum of clinical appearances at presentation, including large ulcerated lesions, large nodular lesions, exceeding 15 cm in diameter. Adjunctive techniques, including biopsy, immunohistochemistry and electron microscopy, can be helpful in questionable cases. In this session, speakers will present the most current data on the clinical presentation, pathology, and management of MCC. Representative and challenging cases will be presented to highlight histopathological diagnosis and treatment options.

To be exact, although MCC lacks specific clinical features, some patients may have constitutional symptoms with evidence of regional or distant metastasis. Heath et al [6] reported AEIOU Features derived from 195 patients of MCC. The biopsy should be considered if the patient presents ≥ 3 features of the above. This study is the first to define the clinical features that may serve as clues in the diagnosis of MCC. With this case, the initial diagnosis was a chalazion, and no histopathologic diagnoses were performed.

The histogenesis of MCC is controversial. Possible cells of origin include the epidermal Merkel cell, a dermal Merkel cell equivalent, a neural-crest-derived cell of the amine precursor uptake. Less commonly, MCC may simulate lymphoma, or may exhibit plasmacytoid, clear cell, anaplastic, or spindle-cell features. Vascular or lymphatic invasion is not uncommon. The tumor in this case showed multi-morphological type such as round, small, plasmacytoid and spindle cells histology. Therefore, this tends to lead to misdiagnosis in some cases, particularly if immunohistochemistry is not performed to confirm the nature of the cells present. In this case, the tumor tissue was positive for CK20, NSE and CAM 5.2, the patient with bad prognostic factors [7,8].CK20 is expressed in a dotlike paranuclear or crescentic pattern. Syn Neurofilament is also expressed in the cytoplasm of most MCC. The above findings support the diagnosis of primary MCC.

Diagnosis of MCC involves the following: General history, physical exam and pathological tests. It is a rare type of skin cancer that is usually misdiagnosed. Although MCC has characteristic clinical features, the diagnosis generally relies on histopathologic identification. Innunohistochemistry is required to differentiate MCC from other small round cell tumors; however, clinical correlation may be required in differentiating MCC from other neuroendocrine tumors that have metastasized to the eyelids. The case we reported was misdiagnosed as chalazion. The exact diagnosis of MCC is made with a biopsy, for special stains are used to distinguish. Immunohistochemistry is very helpful. MCC from other forms of cancer, such as sebaceous cyst, small cell lung cancer (SCLC) and lymphoma, small cell melanoma. Each of these cancers has a unique profile as defined by special stains. CK20 and TTF-1 (positive in SCLC) help distinguish MCC SCLC [9]. Further diagnostic tests are needed, for example, the imaging tests. With this case, the differential histopathological diagnosis should be made with: 1. The tumor in this case showed very large lesion with ulceration and mixed epithelioid and spindle cell histology, and the above presentations may lead to misdiagnosis in some cases [10], particularly if immunohistochemistry is not performed to confirm the nature of the cells present. In our case, we did not see this feature. 2. In this case, the positive assay for CK20, NSE, CAM 5.2 and the negative one for TTF-1and S100. In this tumor, a definition was also supported by multi-metastases in the apex of lung and mediastinal lymphadenovarix of pathological findings on the plain CT chest.

Treatment is generally based on the stage of the disease. There are major treatments for MCC: surgical care and medical care [11]. MCC is chemosensitive but only rarely chemocurable in patients with metastasis or locally advanced tumors. Moreover, a high incidence of toxic death occurs due to chemotherapy. Combination chemotherapy is more effective when two or more drugs are given at the same time because they are more powerful in combination than either individual drug [12]. Primary treatment of the tumor consists of excision with wide margins or micrographic surgery with or without adjuvant radiotherapy. There is a decrease of local recurrence after radiotherapy [13,14]. However, this has no effect on overall survival [15]. Currently, most eyelid MCCs are treated without irradiation. Merkel cell carcinomas respond well to radiation therapy, although some have recurred in the radiation field or during radiotherapy [16]. The goal of wide surgical excision is to control local recurrence and lymph node metastases. MCC should be removed with clear margins as judged by pathology examination. It was recently reported that sentinel lymph nodes was effective in predicting the risk of regional recurrence [17], however, lymph node dissection does not appear to convey a survival advantage [18]. This may be the result of the short follow-up in most reports. There are some reports of responses to interferon [19] and intralesional tumor necrosis factor [20,21]. Radiation therapy, also referred to as radiotherapy, is the treatment of cancer with penetrating beams of energy waves or streams of particles that can destroy cancer cells. Radiation therapy also damages healthy cells in the field of radiation [22]. Cisplatin plus etoposide, cyclophosphamide plus doxorubicin plus vincristine, or cyclophosphamide plus epirubicin plus vincristine are the most commonly used regimens [23]. The response rate is 70%, with a complete response in 35% [24]. Interestingly, nonocular MCC is reported to be a very aggressive tumor, lethal in 33% of patients. In contrast with the literature of MCC at other sites, the authors found only a few patients who died of MCC of the eyelid. This may indicate a good prognosis for eyelid MCC. However, most MCC eyelid studies have a limited follow-up [25]. Overall, the mortality rate is less than 50% in two years, We need more studies including longer-term follow-up.

## Conclusions

In conclusion, this is the first report of a case of MCC with a megalo-neoplasms, high malignance and a poor prognosis. Although reports about MCC have appeared successively, much still remains to be explored about etiological factors, nosogenesis and treatment. It is important to distinguish it from other tumors and early diagnosis and therapy.

## Consent

Informed consent was obtained from the patient for publication of this case report and accompanying images. A copy of the written consent is available for review by the Editor-in-Chief of this journal.

## List of abbreviations

(MCC): Merkel cell carcinoma; (TTF-1): Thyroid transcription factor-1; (CK20): cytokeratin 20, (NSE): neuron specific enolase; leukocyte common antigen (LCA) (MRI): Magnetic resonance imaging; (CT): computerized tomography.

## Competing interests

The authors declare that they have no competing interests.

## Authors' contributions

YJH and BCS proposed the study. LXC and LMZ obtained images and critically write the manuscript provided and reviewed pathological images. JGW and TTL conducted a literature search. All authors read and approved the final manuscript.
